# L-serine at the crossroads of microbiota, intestinal health, and disorders

**DOI:** 10.1038/s42003-026-10133-y

**Published:** 2026-04-28

**Authors:** Amandine Devaux, Delphine Boucher, Romain Villéger, Mathilde Bonnet

**Affiliations:** 1 M2iSH, U1071 Université Clermont Auvergne/Inserm/USC-INRAE 1382, Clermont-Ferrand, France; 2 Ecologie et Biologie des Interactions UMR CNRS 7267, Université de Poitiers, Poitiers, France

**Keywords:** Gastrointestinal diseases, Microbiology

## Abstract

L-serine is an amino acid involved in maintaining intestinal mucosal homeostasis through its role in protein synthesis but also in redox balance, immune function and lipid metabolism. It also contributes to mucus production, the epithelial barrier integrity and gut microbiota composition. Prokaryotic cells, including *Escherichia coli*, have developed mechanisms to exploit L-serine to support their growth, virulence, inflammatory and/or carcinogenic properties. In this review, we summarize current findings on L-serine metabolism in the intestinal mucosal homeostasis, its interactions with microbiota, and its modulation in digestive diseases. Finally, we highlight directions for future research to target L-serine as a promising therapy.

## Abstract (web summary)

In this review, we summarize current scientific findings on L-serine metabolism in the intestinal mucosal homeostasis, its interactions with the gut microbiota, and its modulation in digestive diseases. Finally, we highlight directions for future research to target L-serine as a promising therapeutic strategy.

## Introduction

In the gastrointestinal tract, metabolism is a complex process defined as the totality of chemical and biological transformations involving numerous nutrients, substances, host cells and the microbiota. An imbalance in a metabolite or metabolic pathway can adversely affect intestinal homeostasis, thereby contributing to the development of various pathologies. Amino acids (AA), including L-serine, are among the metabolites most frequently dysregulated. The role of serine in metabolic processes and its interactions with the intestinal microbiota have recently attracted increasing attention. Unlike many amino acids that are primarily used for protein synthesis or as an energy source, serine functions as a central metabolic hub, conferring a unique role in maintaining intestinal cell structure and function. Under stress or pathological conditions, endogenous serine production may be insufficient to meet cellular demands; thus, exogenous serine supplementation may be essential to maintain gut homeostasis. The aim of this review is to summarize current knowledge regarding the influence of L-serine on intestinal homeostasis and its involvement in related pathologies.

## L-serine metabolism in intestinal mucosa

L-serine, first isolated by E. Cramer in 1865, is classified as a nutritionally non-essential AA characterized by the presence of an α-amino group, a carboxyl group, and a hydroxymethyl side chain^1^. The main sources of L-serine in humans are dietary intake, degradation of endogenous proteins, and de novo synthesis. For intestinal cells, this AA originates from either endogenous or exogenous sources.

### Exogenous source

L-serine can be obtained through dietary intake. It is primarily present in meat and shellfish, while higher concentrations are found in eggs and plant-based sources such as soybeans, nuts (almonds, walnuts, peanuts), sesame seeds, chickpeas, and lentils^2–6^. The main dietary source of L-serine is therefore protein, in which L-serine content ranges between 2 and 5%. Lipids, which contain serine in the form of phosphatidylserine (PS) and sphingolipids (SL), are much less important^4–6^. The intestine is the primary site for nutrient absorption and catabolism of L-serine which is released from dietary proteins by the action of enzymes such as pepsin in the stomach, followed by trypsin, chymotrypsin, and carboxypeptidases in the small intestine, and finally by aminopeptidases. L-serine is absorbed in the upper gastrointestinal tract, primarily in the small intestinal mucosa, while a significant portion of unabsorbed L-AA reaches the colon^7,8^. Moreover, it is estimated that 30–50% of AA, including L-serine, is degraded/utilized within the colonic mucosa, and does not enter the bloodstream^9^. In the European Prospective Investigation into Cancer and Nutrition (EPIC) cohort (*n* = 504,245; 147,259 men and 356,986 women), estimated daily dietary L-serine intake falls in the range of approximately 2.6–4.0 g/day. (age- and sex-combined), while the no-observed-adverse-effect-level (NOAEL) for serine was set at 12 g/day of supplementation^4–6^.

Exogenous L-serine is also synthesized by intestinal bacteria, including members of the Pseudomonadota phylum (*Escherichia coli* (*E. coli*), *Salmonella enterica* subsp. *enterica* serovar Typhimurium^10,11^) or Actinomycetota phylum (*Corynebacterium glutamicum*^12^). Bacteria possess the *serACB* operon, which enables them to synthesize L-serine de novo^13–15^. Approximately 15% of the glucose assimilated by bacteria, such as *E. coli*, is utilized for L-serine synthesis^16^. Various strategies, including genetic modifications, have been used to enhance microbial L-serine production^17–21^. Bacteria, mainly from Bacillota (*Eisenbergiella*, *Clostridium XVIII*, *Coprobacillus*), can produce D-serine, the less common enantiomer of L-serine^22,23^. The interconversion of L- and D-AA is catalyzed by a serine racemase (serR), first identified in *Roseobacter litoralis* of the phylum Pseudomonadota^24,25^. This interconversion can also occur in host cells such as enterocytes, neurons and astrocytes. D-serine serves as a key physiological co-agonist of the N-methyl-D-aspartate (NMDA) receptor, a major excitatory neurotransmitter receptor in the central nervous system (CNS)^26,27^. Indeed D-serine has distinct and specialized roles, particularly in neurotransmission. By contrast, L-serine is not a co-agonist of the NMDA receptor; however, it contributes to CNS function by acting as a precursor of D-serine^28^. These functions are beyond the scope of this review and are not further discussed.

### Endogenous source

**Fig. 1:**
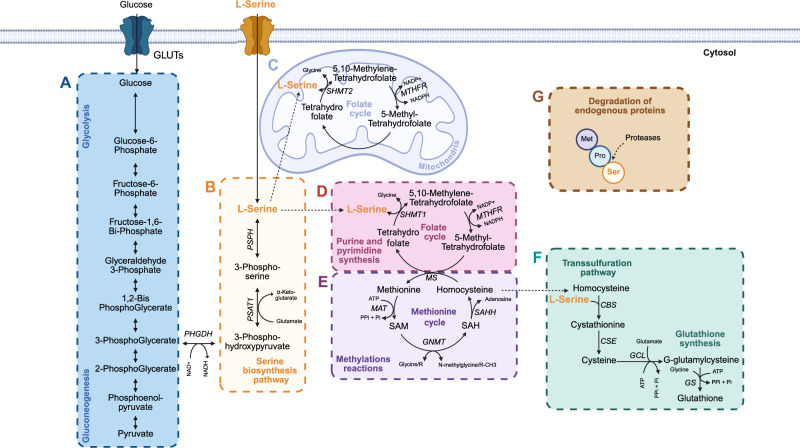
L-serine metabolic pathways in intestinal cells. The serine synthesis pathway occurs in three enzymatic steps (**A**, **B**). First, phosphoglycerate dehydrogenase (PHGDH) catalyzes the NAD⁺-dependent oxidation of 3-phosphoglycerate (derived from glycolysis or gluconeogenesis - **A**) to 3-phosphohydroxypyruvate. Secondly, glutamate-dependent phosphoserine aminotransferase 1 (PSAT1) converts 3-phosphohydroxypyruvate to 3-phosphoserine (**B**). Finally, phosphoserine phosphatase (PSPH) hydrolyzes 3-phosphoserine to produce L-serine (**B**). The interconversion between L-serine and glycine integrates it into the folate cycle (mitochondria and/or cytosol) (**C**, **D**) and subsequently into the methionine cycle (**E**), contributing to purine nucleotide synthesis and methylation reactions, respectively. Additionally, L-serine serves as a precursor for H_2_S and cysteine synthesis, which is further utilized in the biosynthesis of glutathione *via* the transsulfuration pathway (**F**). L-serine can also be obtained through the proteolytic degradation of endogenous proteins (**G**). Abbreviations: ATP Adenosine triphosphate, CBS Cystathionine beta synthase, CSE Cystathionine gamma-lyase, GLUT Glucose transporter, GCL Glutamate cysteine ligase, GNMT Glycine N-methyltransferase, GS Glutathione synthase, H_2_S Hydrogen sulfide, MAT Methionine adenosyltransferase, MTHFR 5,10-Methylenetetrahydrofolate reductase, MS Methionine synthetase, Oxidized nicotinamide adenine dinucleotide (NAD^+^), NADH Reduced nicotinamide adenine dinucleotide, NADPH Reduced nicotinamide adenine dinucleotide phosphate, PHGDH Phosphoglycerate dehydrogenase, Pi Inorganic phosphate, PPi Inorganic pyrophosphate, PSAT Phosphoserine aminotransferase 1, PSPH Phosphoserine phosphatase, SAM S-adenosylmethionine, SAH S-adenosyl-L-homocysteine, SAHH S-adenosyl-L-homocysteine hydrolase, SHMT Serine hydroxymethyltransferase.

Serine can be synthesized endogenously when dietary intake is insufficient. L-serine is produced by intestinal cells *via* three main pathways: de novo synthesis through the serine synthesis pathway (SSP), conversion from glycine through the folate-dependent one-carbon cycle, and protein degradation (Fig. 1)^29^. The SSP begins from 3-phosphoglycerate (3PG) obtained from glycolysis or gluconeogenesis and proceeds in three steps: oxidation of 3PG to 3-phosphohydroxypyruvate (3PHP) by Phosphoglycerate dehydrogenase (PHGDH), transamination to 3-phosphoserine (3PSer) by phosphoserine aminotransferase 1 (PSAT1) and hydrolysis to L-serine by phosphoserine phosphatase (PSPH)^30^ (Fig. 1A, B). PHGDH, the first SSP enzyme, is markedly upregulated in HCT-116 colon cancer cells, in contrast to normal colonic epithelial cells, which exhibit only basal levels of expression.^31^. The SSP is active in endothelial cells^32^, mucus-secreting goblet cells^33^ and immune cells^34,35^, underscoring its importance in intestinal mucosal cell metabolism. However, its role in Paneth cells and enteroendocrine cells remains unexplored. This pathway is inhibited by an excess of serine and is tightly regulated by growth factors and nutritional cues. In the fed state, glucose is the primary substrate fueling the SSP, whereas during fasting, neoglucogenesis contributes to approximately 70% of L-serine synthesis^36^. PHGDH is the rate-limiting step of the pathway, and it is often overactivated in certain cancers and rapidly proliferating tissues.

L-serine can also be generated through the folate cycle *via* serine hydroxymethyltransferase (SHMT) 1 localized in the cytoplasm (Fig. 1C) and SHMT2 in the mitochondria (Fig. 1D)^37^. SHMT enzymes play a pivotal role in cellular one-carbon metabolism by catalyzing the reversible conversion of glycine to L-serine and coupled to the interconversion of 5,10-methylenetetrahydrofolate (5,10-CH2-THF) and tetrahydrofolate (THF). SHMT has a bacterial homolog encoded by the *glyA* gene^38^. In bacterial cells, as in eukaryotic cells, SHMT plays a central role in glycine and L-serine metabolism. Protein degradation represents a significant source of intracellular L-serine in the intestinal mucosa. The intestinal epithelium undergoes intense protein turnover, with approximately 50% of mucosal proteins being renewed daily in humans, reflecting continuous synthesis and degradation cycles that contribute to cellular amino acid pools^39^. Protein degradation is mediated by multiple intracellular systems, including the ubiquitin–proteasome pathway and lysosome-dependent autophagy. The amino acids liberated by intracellular proteolysis—including L-serine—can be reassimilated into metabolic pathways to support cellular demands under conditions of limited nutrient supply or increased metabolic demand.

### Integration of L-Serine into intestinal cellular metabolic pathways

L-serine acts as a precursor for protein synthesis, and contributes to pyruvate production for gluconeogenesis, thereby sustaining basal cell metabolism^40^. It is also essential for lipid biosynthesis, including the generation of sphingolipids, phosphatidylserine, and phosphatidylethanolamine, which are crucial for cell signaling and membrane integrity in eukaryotic cells^41^. L-serine plays a key role in one-carbon metabolism through its involvement in the synthesis of other AA^42^. Beyond its structural and energetic roles, L-serine is a central contributor to one-carbon metabolism. Through the reversible action of serine hydroxymethyltransferases SHMT1 and SHMT2, L-serine is converted to glycine while donating a one-carbon unit to THF, resulting in the formation of 5,10-methylene-THF. This carbon unit feeds into the folate cycle and, together with the methionine cycle, supports methyl-group generation and nucleotide biosynthesis (Fig. 1C–E). This process is required for de novo purine synthesis and cell growth^43^.

L-serine also contributes to the transsulfuration pathway, leading to the production of cysteine and subsequently glutathione, a key antioxidant involved in maintaining cellular redox homeostasis^44^. In parallel, this pathway allows the generation of hydrogen sulfide (H₂S), a metabolite that contributes to gastrointestinal homeostasis.

In conclusion, the major routes of intracellular L-serine utilization and degradation include its conversion to glycine and its metabolism through the transsulfuration pathway (Fig. 1F). When intracellular L-serine levels are elevated, only a fraction is converted to glycine, whereas the remainder is directed toward folate-dependent reactions and protein biosynthesis.

### Transporters

Following dietary intake and/or endogenous synthesis by the intestinal microbiota and epithelial cells, L-serine becomes available to various components of the digestive tract through multiple eukaryotic transporter systems^45^. Extracellular L-serine can be imported into intestinal epithelial cells through specialized solute carrier (SLC) transmembrane transporters, which mediate the uptake of neutral amino acids including L-serine. Conversely, L-serine can also be exported *via* certain SLC transporters which facilitates amino acid exchange and contributes to local serine homeostasis.

The first SLC transporters is the Alanine Serine Cysteine (ASC) system, a sodium-dependent transporter responsible for the uptake of neutral AA including alanine, cysteine, and serine. It comprises two principal transporters: Alanine Serine Cysteine Transporter (ASCT) 1, encoded by the *slc1a4* gene, and ASCT2, encoded by the *slc1a5* gene. ASCT1 is expressed ubiquitously with especially high levels in the brain, skeletal muscle, and pancreas whereas ASCT2 is predominantly expressed in the kidney, colon, lung, skeletal muscle, testis, and adipose tissue^42^. The second system is the System A transporters, which mediate the sodium-dependent uptake of small neutral amino acids, with a preference for alanine. Key members of this system sodium-coupled neutral AA transporter (SNAT) include SNAT1 (SLC38A1) and SNAT2 (SLC38A2), which are widely expressed in numerous organs, including the bladder, kidney, pancreas, liver, and the digestive tract^46^. In addition, the sideroflexin 1 transporter interacts with the Serine Active Site Containing 1 protein on the outer mitochondrial membrane to facilitate the import of L-serine into mitochondria and support the activation of one-carbon metabolism^47,48^. The coordinated activity of these transporters, together with intracellular biosynthesis and protein degradation, ensures that epithelial cells maintain adequate L-serine levels to support their high metabolic and proliferative demands.

Prokaryotic systems also possess specific L-serine transporters. Notably, the *tdcC* gene, located within the *tdc* operon, and the *sdaC* gene, part of the *sdaCB* operon, are involved in L-serine uptake and catabolism in bacteria^49–52^.

## Role of L-serine in intestinal mucosa homeostasis

**Fig. 2:**
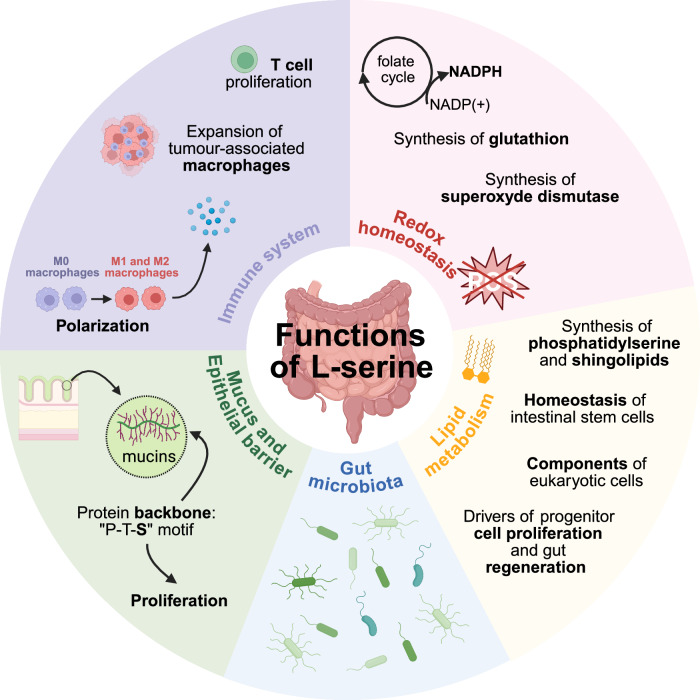
Involvement of L-serine in intestinal homeostasis. L-serine is involved in intestinal homeostasis, *via* its contribution to the immune system, redox homeostasis, lipid metabolism, mucus and epithelial barrier and gut microbiota. Abbreviations: “P-T-S” (Proline-Threonine-Serine), NADP(+) (Oxidized nicotinamide adenine dinucleotide phosphate), NADPH (Reduced nicotinamide adenine dinucleotide phosphate).

The extensive involvement of L-serine in numerous metabolic pathways underscores its central role in intestinal homeostasis, influencing a wide range of processes including redox homeostasis, lipid metabolism, immune responses, mucus synthesis, epithelial barrier integrity, and gut microbiota function (Fig. 2).

### L-serine and protein functions

Due to its polar side chain, L-serine is predominantly located on the surface of proteins, where it contributes to hydrophilic interactions between proteins and other molecules. L-serine residues are important for regulating protein function because they are part of the O-glycosidic bonds of glycoproteins and constitute a primary site of protein phosphorylation. Moreover, L-serine is found in the catalytic sites of hydrolytic enzymes^53^. Through its structural and regulatory roles in proteins, L-serine is essential for maintaining intestinal mucosal integrity.

### Mucus synthesis and epithelial barrier

Mucus is a viscoelastic gel predominantly composed of approximately 95% water and 1–5% mucins, which function as key physical and chemical barriers against bacterial invasion within the intestinal lumen. The mucus matrix is primarily formed by mucin glycoproteins, characterized by repetitive domains enriched in proline-threonine-serine (P-T-S) motifs that serve as principal sites for O-linked glycosylation and form the common biophysical structure of mucins, such as Muc2^54,55^. These AA-enriched residues facilitate the transfer of N-acetylgalactosamine, initiating the synthesis of mucin oligosaccharides^56^. These processes occur in the Golgi apparatus of goblet cells in the colon, before the products are secreted^33,57^. This extensive glycosylation critically modulates the structural integrity and functional properties of the mucus layer^58^. Supplementation with AA, including L-serine, has been shown to stimulate mucin synthesis in the colon, marked by an increased number of Muc2-containing goblet cells in Dextran-Sulfate-Sodium (DSS) - treated rats^59^. Dietary L-serine supplementation significantly increases *Muc2* mRNA expression in the ileal mucosa of laying hens compared to controls^60^. Serine supplementation improves villus morphology in weaned piglets^61^ and preserves barrier integrity, reduces permeability, and promotes epithelial healing in DSS-treated mice^62,63^. Certain bacteria of the intestinal microbiota can degrade serine found in mucus, facilitating their ability to colonize the intestinal epithelium^64^. Consequently, L-serine represents a major amino acid constituent of the mucus, underpinning its essential role in mucosal barrier architecture and function.

### Redox homeostasis

L-serine supports intestinal homeostasis by maintaining cellular redox balance, primarily through its contribution to the synthesis of the reducing agent nicotinamide adenine dinucleotide phosphate (NADPH) *via* the folate cycle and to glutathione synthesis, a major cellular antioxidant^44,65–67^.

Serine deficiency exacerbates oxidative stress, reducing antioxidant enzyme activity and glutathione levels in aging mice and intestinal porcine epithelial cells (IPEC-J2 cells)^68,69^. Similarly, depletion of *shmt2* promotes the accumulation of reactive oxygen species (ROS) by disrupting cellular redox status, although this effect was observed in bladder cells^70^.

In contrast, L-serine supplementation enhances antioxidant defenses by increasing glutathione synthesis and reducing ROS in various models, including IPEC-J2 cells and diquat-treated mice (a well-known oxidative stress inducer)^69,71^. Long-term L-serine administration increases superoxide dismutase and glutathione levels, thereby alleviating oxidative stress in aging mice^72^. Similarly, in pigs, dietary serine improves antioxidant capacity by decreasing malondialdehyde levels while increasing glutathione and superoxide dismutase activities^73^. Moreover, maternal serine availability is critical for maintaining antioxidant defenses against oxidative stress in weaned mouse offspring^74^.

### Lipid metabolism

L-serine is involved in the de novo synthesis of phosphatidylserine and sphingolipids (SL). SL formation begins with the condensation of L-serine and palmitic acid by serine palmitoyltransferase (SPT), the first and rate-limiting enzyme of this pathway. SPT catalyzes the condensation of L-serine with palmitoyl-coenzyme A (CoA) to form 3‑ketodihydrosphingosine (also known as 3‑ketosphinganine), which is subsequently reduced and modified to produce ceramide - the central hub of sphingolipid metabolism^41,75,76^. Mammalian SPT is a heterodimeric enzyme composed of the LCB1 and LCB2 subunits, anchored to the endoplasmic reticulum membrane, and requires pyridoxal‑5′‑phosphate (PLP) as a cofactor^75^. This step is crucial because it dictates the composition of long‑chain bases that define different sphingolipid species. The unavailability of L-serine following depletion of *phgdh* gene, low serine levels or SPT mutation, leads to a reduction in SL levels. Under these conditions, SPT utilizes alanine as a substrate instead of serine. This alternative substrate usage results in the accumulation of 1-deoxySL, which lack the canonical C1-hydroxyl group required for further metabolic processing. These deoxySL, including 1-deoxydihydroceramide and 1-deoxyceramide, are cytotoxic and cannot be efficiently degraded or incorporated into normal sphingolipid pathways^77^. Thus, L-serine critically regulates SPT activity and sphingolipid homeostasis, with significant implications for cell viability, progenitor cell proliferation and intestinal regeneration by fueling sphingolipid synthesis^78^. Moreover, these results emphasize that de novo synthesis of L-serine is necessary to maintain SL homeostasis^79,80^.

Sphingolipids are critical structural and signaling components of epithelial membranes, where they help maintain barrier integrity, regulate cell polarity and mediate turnover and repair. The excision of an SPT subunit, *Sptlc2*, in intestinal stem cells (ISCs) leads to disruption of intestinal architecture and a reduction in the proliferation of these cells^81^. Similarly, inhibition of SL synthesis in *Drosophila melanogaster* impairs ISCs proliferation, highlighting that SL are key drivers of progenitor cell proliferation and gut regeneration^78^. Beyond host cells, SL are also synthesized by certain bacteria^82^. These findings are discussed in detail in the section “*Interaction with the gut microbiota*” below.

### Immune regulation and inflammation

L-serine plays a critical role in the immune response by supporting cytokine production and macrophage polarization.

In peritoneal and bone-marrow-derived macrophages (BMDM), L-serine promotes Interleukin (IL) 1β expression *via* histone H3 modifications (H3K36me3, H3K9/27 acetylation), and activation of the nucleotide-binding domain and leucine-rich repeat containing proteins 3 (NLRP3) inflammasome^34,83,84^. Serine deprivation reduces anti-inflammatory IL-10 and increases pro-inflammatory IL-6^85^.

Serine metabolism also plays a significant role in M1 or M2 macrophage polarization^86–88^. M1 macrophages exhibit bactericidal, pro-inflammatory, and anti-tumor functions, whereas M2 macrophages are primarily involved in tissue repair and anti-inflammatory processes and pro-tumor activities^88^. L-serine contributes ex vivo to mouse peritoneal M1 macrophage polarization, through mechanistic target of rapamycin (mTOR)^89^ or histone H3K36 trimethylation^83^. Consequently, endogenous or exogenous L-serine restriction affects the polarization of lipopolysaccharide (LPS) - activated M1 macrophages isolated from mouse peritoneum or BMDM^84^. However, contrasting findings suggest that serine restriction may enhance interferon (IFN) - γ-induced M1 polarization both in vitro and in vivo^90^. Serine is also crucial for M2 polarization, as PHGDH is associated with M2 macrophage polarization derived from BMDMs^90,91^. However, most of these findings derive from non-intestinal macrophage models.

L-serine also plays a key role in regulating T cell proliferation and function^35,92^. It is incorporated into effector T cells supporting their expansion during *Listeria* infection in vivo^93^. Serine biosynthesis *via* PHGDH is essential for CD8 + T cell proliferation, while SHMT2 is required for T cell survival^94,95^. Dietary serine restriction impairs antigen-specific T cell responses. However conflicting results have been reported in mouse models of colorectal cancer (CRC)^96,97^, which will be discussed in the final section.

### Interaction with the gut microbiota

Several studies have demonstrated that L-serine supplementation or restriction can influence gut microbiota composition. Dietary L-serine supplementation modulates the composition of gut microbes in a pig model, as evidenced by an increase in the relative abundance of *Streptococcus* and *Lactobacillus* and a decrease in the relative abundance of *Clostridium_sensu_stricto_1* and *Terrisporobacter*. The enriched microbial metabolites (acetylcarnitine, ethylmalonic acid, nonanoic acid, N8-acetylspermidine and L-aspartic acid) were mostly associated with lipid synthesis, confirming the link between L-serine and lipid metabolism^73^. Conversely, dietary L-serine deficiency disrupts gut microbiota composition, characterized by reduced microbial diversity and a decreased Bacillota/Bacteroidota ratio in aging-mice^68^. L-serine serves as an important source of energy and nitrogen for the intestinal microbiota. Experiments using labeled carbon have shown that bacteria can utilize serine to generate pyruvate and acetyl-CoA^98–100^. L-serine has been shown to support the growth of various bacteria, including *E. coli*, *Klebsiella aerogenes* and *Streptococcus sp* in semi-defined medium^101^. In contrast, L-serine can inhibit the growth of certain bacterial strains and enhance the efficacy of antibiotic treatment^102,103^. For example, the addition of L-serine restored antibiotic efficacy against *Streptococcus suis* by promoting intracellular H_2_S production and disrupting iron-sulfur clusters, leading to increased ROS, DNA damage, and bacterial death^104^. Although *S. suis* is not an intestinal species, this finding highlights the dual role of L-serine in modulating bacterial growth and metabolism across diverse bacterial taxa.

**Fig. 3:**
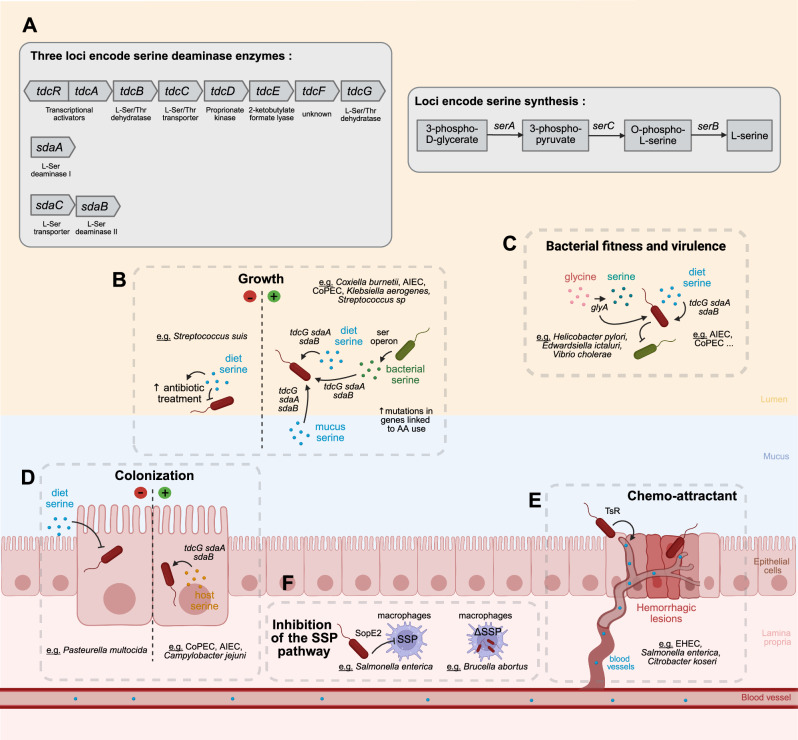
Role of L-Serine in intestinal microbiota dysbiosis and bacterial virulence. L-serine from diet, bacteria, the proline-threonine-serine motifs of the mucin backbone and host cells can be utilized by bacteria *via* serine deaminases (**A**). This provides several advantages, including enhanced bacterial growth (**B**), bacterial fitness and virulence (**C**) and intestinal colonization (**D**). Virulence can also be acquired through the synthesis of L-serine from glycine *via* the *glyA* gene (**C**). Certain bacteria have developed a serine chemoreceptor (TsR) that degrade mucus and direct bacteria toward enterohaemorrhagic lesions (**E**). However, some studies have reported that L-serine supplementation can inhibit bacterial growth and colonization (**B**, **D**). Additionally, specific L-serine-sensitive-bacteria can suppress the SSP in macrophages, via the type III secretion system effector protein, SopE2, thereby promoting their own growth (Panel F). *Brucella abortus* exhibits enhanced growth within macrophages depleted of the L-serine biosynthesis pathway (ΔSSP) (Panel F). Abbreviations: AA Amino acids, AIEC Adhesion-invasive *Escherichia coli*, CoPEC Colibactin-producing *Escherichia coli*, EHEC Entero-hemorrhagic *Escherichia coli*, SopE2 Type III secretion system effector protein, SSP L-serine synthesis pathway.

Interestingly, an operon, named “tdc”, allows bacteria to catabolize L-serine and convert it into pyruvate and ammonia^49–51^ (Fig. 3). This operon consists of several genes, including the L-serine dehydratase gene (*tdcG*)^105^, and also enables the degradation of L-threonine^106^. Few studies have investigated the prevalence or expression of this operon in the colon of mice or humans. According to MetaQuery platform, the transcriptional activators of the tdc operon (*tdcA*-K07592; *tdcR*-K07591), are prevalent in 93.25% and 38.73% of the 1267 fecal metagenomes analyzed from healthy individuals and those suffering from various pathologies (colorectal cancer, Crohn’s disease (CD), obesity, diabetes, and rheumatoid arthritis)^107^. No data concerning *tdcG* are known^107^. Two additional enzymes for degrading L-serine are know: L-serine deaminase I (*sdaA*) and L-serine deaminase II (*sdaB*)^108,109^ (Fig. 3). The latter is located on the sdaCB operon, which also encodes an L-serine transporter (*sdaC*)^52^. According to MetaQuery platform, *sdaA*-K01752 and *sdaC*-K03837 are detected in 100% and 78,07% of the 1267 fecal metagenomes analyzed, respectively. No information is available for *sdaB*^107^. The genes involved in degradation (*tdcG*, *sdaA* and *sdaB*) share a similar structure, with 71% to 75% identical in their desoxyribonucleic acid (DNA) sequence. These genes are notably found in Pseudomonadota, such as in the genome of *Escherichia coli*, *Citrobacter koseri*, *Salmonella enterica* or *Proteus mirabilis*. In *E. coli* K12, the expression of these genes is differentially regulated by environmental conditions. Specifically, *sdaA* exhibits higher expression in minimal media, whereas *sdaB* and *tdcG* are enhanced in nutrient-enriched media (except in the presence of glucose). Furthermore, *sdaA* and *sdaB* expression is increased under aerobic conditions, while *tdcG* is upregulated under anaerobic conditions^110^.

As mentioned in section 2.4. certain gut bacteria—particularly those belonging to the Bacteroidota phylum—can produce SL from L-serine. SL play an essential role in maintaining intestinal homeostasis by shaping immune tolerance and modulating inflammatory responses. Studies using SL-deficient *Bacteroides thetaiotaomicron* mutants have demonstrated that the loss of bacterial SL synthesis results in heightened gut inflammation, altered host ceramide profiles, and impaired epithelial barrier integrity^111^. SL derived from *Bacteroides* can be incorporated into host lipid metabolic pathways and function as signaling molecules, interacting with host receptors such as Toll-like receptor 2 to influence macrophage activation and cytokine production. Together, these findings highlight microbial SL as key mediators of host–microbiota communication, linking metabolic regulation, immune responses, and maintenance of the epithelial barrier.

## Involvement of L-serine in intestinal pathologies

Serine is defined as a non-essential amino acid. Due to its critical role in intestinal mucosa homeostasis, dysregulation of L-serine metabolism is associated with numerous intestinal pathologies, such as Inflammatory Bowel Diseases (IBD) including CD and Ulcerative Colitis, or CRC. A serine-depleted (SD) diet in wild-type (WT) mice does not seem to affect food intake or significantly alter body weight or colon length^112–115^. Recently, it was found that under pathological conditions, endogenous L-serine is insufficient. Isotopic tracer experiments have shown high L-serine synthesis from labeled glucose in rapidly proliferating cells, such as cancer cells, indicating active biosynthesis^27^. Thus, elevated levels of L-serine have been detected in the feces and serum of CRC patients^116–120^. Similarly, in IBD, serine synthesis is increased in the colonic epithelium of patients^62^. These findings suggest the importance of serine metabolism in intestinal pathophysiology.

### Epithelial cell proliferation

Cells can acquire serine either through the SSP or by importing it from the extracellular environment *via* eukaryotic L-serine transporters^121^. ASCT2 is overexpressed in colorectal adenomas^122^, and SLC6A14 and SLC25A15 have been identified as key transporters supporting CRC cell proliferation^123^. Tumors are dependent on extracellular L-serine, such as HCT116^113^, RKO and SW480 colon cancer cells^124^, particularly in p53-mutant cancer cells^113^. In contrast, intestinal tumor organoids harboring KRAS mutations are less dependent on extracellular L-serine, compensating instead by upregulating the SSP^125^. Consistently, feeding L-serine-containing diets to CT26- and HCT116-tumor-bearing mice as well as APC^min/+^ mice, led to increased tumor growth^77,113,125^. The SSP is frequently implicated in digestive cancers and IBD, primarily because of its role in supporting cell proliferation. Elevated PSAT expression and increased serine levels have been observed in the colons of patients with IBD and in DSS-treated mice, where they promote mucosal regeneration by enhancing wound healing and crypt cell proliferation^62^. Overexpression of the *phgdh* gene has been observed in colon^126^ and gastric cancers^127^, and is associated with poor prognosis, higher tumor grade and larger tumor sizes in CRC^128^. Moreover, PHGDH promotes colon cancer metastasis by increasing *S*-adenosylmethionine levels and activating the expression of the cell adhesion-related genes^129^. SHMT is likewise upregulated in multiple cancers and serves as a predictor of unfavorable clinical outcomes^130–133^. In esophageal squamous cell carcinoma (ESCC), serine metabolism supports tumor cell proliferation by promoting purine nucleotide synthesis^134^. Although ESCC originates from the upper digestive tract and its metabolic features cannot be directly extrapolated to intestinal cancers, these findings suggest that serine deprivation may suppress tumor cell proliferation by limiting purine nucleotides and NADPH synthesis.

### Lipid metabolism

As outlined above, L-serine is a component in sphingolipid biosynthesis *via* the SPT enzyme. The SL biosynthesis pathway and SPT are over-expressed in human intestinal adenomas^78^. Increased production of SL, including ceramide, stimulates Fatty Acid-Binding Protein 1, thereby enhancing the uptake of saturated fats. This mechanism promotes the proliferation of ISCs, a process leading to the accumulation of somatic mutations and increasing the risk of benign adenomas progressing to CRC^78^. A second study demonstrated a correlation between the *psat1* gene overexpression and increased production of ceramide-derived extracellular vesicles in CRC^135^. Furthermore, metabolomic analysis of 630 metabolites present in the tumor microenvironment revealed elevated levels of serine and palmitic acid, which are essential for sphinganine biosynthesis. Moreover, this lipid promotes the differentiation of Treg cells and thus contributes to carcinogenesis (see next section)^136^.

### Redox homeostasis, immunity, and intestinal inflammation

L-serine contributes to the maintenance of redox balance in ESCC cells by participating in NADPH synthesis, which is crucial for preserving redox homeostasis, preventing ROS-induced DNA damage and maintaining intestinal homeostasis^134^. Similarly, L-serine is an important driver of inflammation in IBD, through its effects on mitochondrial function and macrophage proliferation^62^. The SSP pathway sustains immunosuppressive M2 macrophage activation and TAM expansion in tumor-bearing mice^137^. An SD diet exacerbates weight loss, reduces colon length, and increases stool bleeding and diarrhea in models of DSS-induced colitis and in *shPsat1* mice (short hairpin RNAs, shRNAs targeting the 2nd gene in the SSP pathway), consistent with worsened disease severity and inflammation^62,114^. Conversely, 0.2% serine supplementation increases body weight gain and reduces diarrhea incidence in weaned piglets^61^. Saha et al. and Tong et al. highlighted the link between L-serine and the suppression of anti-tumor immunity^96,97^. L-serine inhibits cGAS-STING signaling, which is involved in type I IFN production and the intratumoral infiltration of immune cells (dendritic, CD4⁺ and CD8⁺ T cells)^96^. Furthermore, L-serine influences anti-tumor immunity through sphinganine synthesis. Sphinganine interacts with the transcription factor c-Fos, inducing the transcription of target genes, such as *Pdcd1* which promotes the differentiation of regulatory T cells and thereby suppresses anti-tumor immunity^136^.

### Microbiota dysbiosis and bacterial virulence

Microbiota dysbiosis described in digestive pathology could affect L-serine availability for intestinal cells impairing intestinal mucosa homeostasis^49,52^. Patho-adaptative alterations in bacterial genes associated with AA metabolism provide some bacteria with a competitive growth advantage over non-pathogenic strains^138^. Moreover, L-serine may be an AA that influences the virulence of certain bacteria, potentially leading to dysbiosis and/or disrupting intestinal homeostasis. L-serine in serum is recognized by the TsR bacterial chemoreceptor, attracting Enterobacteriaceae and enables them to provide a competitive advantage for migration into enterohaemorrhagic lesions^139^ (Fig. 3). In the inflamed intestine, L-serine promotes the expansion of adherent-invasive *E. coli* (AIEC, pathotype of *E. coli* isolated from CD patients), highlighting a link between L-serine and the microbiota in intestinal pathologies^112^. Moreover, it has been described that AIEC shifts its metabolism to catabolize L-serine in the inflamed gut, thereby enhancing bacterial fitness in the inflamed environment (Fig. 3). Indeed, the tdc operon is strongly up-regulated in the inflamed gut of germ-free mice infected with AIEC and treated with DSS^112^. Other bacteria, such as *Campylobacter jejuni*, responsible for diarrhea, catabolize L-serine to colonize the digestive tract^140^. A deficiency in bacterial genes involved in L-serine synthesis has been demonstrated to reduce the survival and proliferation of *Brucella abortus* in macrophages^11^. Infection with *Salmonella enterica* subsp. *enterica* serovar Typhimurium, invasive enteric pathogen, represses serine synthesis in macrophages in vitro, *via* a type III secretion system effector protein called SopE2, leading to the accumulation of 2-PG, 3-PG and phosphoenolpyruvate, which serve as carbon sources^141^ (Fig. 3). A *Helicobacter pylori* deficient for *glyA* gene exhibits markedly slowed growth and loss of the virulence factor CagA^38^. In the same way, Dahal et al. show that a *glyA* deficient strain of *Edwardsiella ictaluri* presents a significant attenuated virulence. *GlyA1* gene has been identified as a new virulence factor, required by *Vibrio cholerae* to colonize the infant mouse intestine^142^.

In CRC, L-serine also plays a multifaceted role through gut microbiota. Serine levels differ significantly in fecal samples between adenoma patients and controls prior to endoscopy, as well as in adenoma patients before and three months after polypectomy. These variations correlate with the genera *Faecalitalea* and *Butyricimonas* in patients with adenomas compared to controls, suggesting that alterations in AA levels may result from, or contribute to, specific changes in gut microbiota composition^143^. Recently, Devaux et al. demonstrated that L-serine promotes pro-carcinogenic effects of colibactin-producing *E. coli*. These bacteria use the host’s L-serine, so as to persist and promote its genotoxic and pro-inflammatory effects in the colon^144^.

While further investigations are necessary to fully elucidate the interplay between serine, the microbiota, and the intestinal mucosa, this amino acid emerges as a key modulator of host–microbiota interactions implicated in intestinal pathologies.

### Targeting L-serine in the management of intestinal diseases

Given L-serine’s role in intestinal homeostasis and its dysregulation in diseases, studies have investigated its supplementation or depletion, primarily through dietary interventions. In inflammatory models, AA supplementation including L-serine, restores intestinal barrier homeostasis by increasing mucin synthesis in the colon and improving colonic morphology, accompanied by beneficial shifts in microbial composition, such as increased *Enterobacteriaceae*, *Enterococcus* and *Lactobacillus*^59,145^. These findings are consistent with the study by Bai et al., which highlighted the role of L-serine in colonic mucosa regeneration following injury in a DSS-induced colitis model^62^. In a similar model, rectal supplementation with L-serine induces modifications in bacterial community structure, characterized by an increase of OTUs (Operational Taxonomic Unit), the phylum Bacillota, the orders Clostridiales and Lactobacillales, and a reduction in the phylum Bacteroidota^145^. Conversely, serine depletion worsens acute DSS-induced injury and impairs wound healing^62^. Clinically, oral L-serine has shown good tolerability in early studies targeting neurological conditions such as amyotrophic lateral sclerosis and sensory neuropathies, although efficacy remains limited and further investigation is warranted^146,147^. However, recent studies have reported conflicting results using L-serine-depleted diets, highlighting the complex relationship between L-serine and IBD. In germ-free mice colonized with CD microbiota, a SD diet reduced inflammation and pathogenic AIEC colonization, although AIEC may adapt by sourcing host epithelial cells-derived serine^112,114^. In digestive cancers, targeting L-serine *via* a SD diet^77,113,125,136^, transporter blockade (e.g., SLC6A14/SLC25A15)^123^, or inhibition of the SSP^77,135,148–152^ impairs tumor growth in several preclinical models. However, more recent studies have reported contradictory findings such as no significant reduction in the growth of HCT116, DLD1 or CT26 grafts under an SD diet^115,153,154^. It has been hypothesized that cancer cells can compensate for L-serine deprivation by acquiring it from alternative sources or *via* SSP upregulation^155^, highlighting the need for combined strategies to reduce tumor development^115,153^. Moreover, targeting L-serine metabolism enhances the response to standard anti-cancer treatments. Van de Gucht et al. demonstrated that SSP inhibition increased the sensitivity of hypoxic CRC cells to radiotherapy involving mitochondrial tricarboxylic acid (TCA) cycle dysfunction, and consequent ROS overproduction^156^. Dysregulation of the TCA cycle also disrupts pyrimidine synthesis, which is essential for cancer cell proliferation^157^. L-serine depletion maximizes the efficacy of 5-Fluorouracil (5-FU), a chemotherapy used in CRC^153,154^. Resistance to 5-FU has been associated with increased dependence on L-serine, either through SSP upregulation or enhanced serine uptake. Serine-driven mitochondrial metabolism contributes to 5-FU resistance by supporting purine nucleotide biosynthesis, thereby facilitating the repair of chemotherapy-induced DNA damage^154^. Furthermore, L-serine depletion promotes response to immunotherapies by increasing CD4+ and CD8 + T cell infiltration and activating the cGAS-STING pathway *via* mitochondrial DNA leakage^96,97^. Additionally, Ke et al. demonstrated in a *Caenorhabditis elegans* model that interactions between L-serine and the gut microbiota modulate chemotherapy responses^158^.

SD diets are rarely described in clinical studies, as L-serine deficiency can lead to metabolic and functional alterations. Implementing an SD diet is challenging due to the ubiquitous presence of L-serine in dietary proteins. In cancer patients, Buqué et al. have suggested combining a free- or low-protein diet with AA supplementation excluding serine. This strategy must consider the potential adverse effects of serine depletion and may not be suitable for all patients, particularly those with cachexia or in pediatric populations^159^. To date, only one phase I study has investigated the impact of an SD diet in advanced cancer patients (20 individuals, 80% with digestive cancer), focusing primarily on safety rather than efficacy. The diet included a serine-free nutrient powder and low-amino acid fruits and vegetables. The study reported no significant toxicity, apart from gastrointestinal symptoms (diarrhea, nausea, and flatulence) which were managed symptomatically with minimal impact on quality of life^97^. These symptoms may also be influenced by tumor development in the digestive tract. This trial showed partial responses or disease stabilization in most patients, with promising associations between serum serine levels, immune activity, and tumor response, suggesting that this diet approach may enhance immunity and inhibit tumor development. Among patients with CRC, 10% exhibited a partial response, 80% achieved stable disease, and 10% experienced disease progression^97^. Further research is required to assess the toxicity of SD diet or SSP inhibitor, and to evaluate the impact of serine depletion on tumor progression. To date, no PHGDH inhibitors have been tested in clinical studies, and concerns remain regarding toxicity, particularly in the CNS and during development^160^.

## Conclusion

Although defined as non-essential, L-serine plays critical roles in redox homeostasis, lipid metabolism, immune function, and mucosal defense against pathogens, highlighting its importance in maintaining intestinal homeostasis. While further studies are needed to clarify the interactions between L-serine, microbiota and the intestinal mucosa, these findings collectively underscore the potential of therapeutic strategies targeting serine metabolism, such as microbial modulation and/or specific dietary interventions, particularly in IBD and CRC.

### Reporting summary

Further information on research design is available in the Nature Portfolio Reporting Summary linked to this article.

## Supplementary information

Reporting Summary
